# Estimation of Soil Organic Matter in Arid Zones with Coupled Environmental Variables and Spectral Features

**DOI:** 10.3390/s22031194

**Published:** 2022-02-04

**Authors:** Zheng Wang, Jianli Ding, Zipeng Zhang

**Affiliations:** 1 Xinjiang Key Laboratory of Oasis Ecology, Xinjiang University, Urumqi 830046, China; wz_wangzheng@stu.xju.edu.cn (Z.W.); zp_zhang@stu.xju.edu.cn (Z.Z.); 2 Key Laboratory of Smart City and Environment Modelling of Higher Education Institute, School of Geographical Sciences, Xinjiang University, Urumqi 830046, China

**Keywords:** remote sensing, visible and near-infrared spectroscopy, soil organic matter, environmental variables, partial least squares regression

## Abstract

The soil organic matter (SOM) content is a key factor affecting the function and health of soil ecosystems. For measurements of land reclamation and soil fertility, SOM monitoring using visible and near-infrared spectroscopy (Vis-NIR) is one approach to quantifying soil quality, and Vis-NIR is important for monitoring the SOM content in a broad and nondestructive manner. To investigate the influence of environmental factors and Vis-NIR spectroscopy in estimating SOM, 249 soil samples were collected from the Werigan–Kuqa oasis in Xinjiang, China, and their spectral reflectance, SOM content and soil salinity were measured. To classify and improve the prediction accuracy, we also take into account the soil salinity content as a variable indicator. Relevant environmental variables were extracted using remote sensing datasets (land-use/land-cover (LULC), digital elevation model (DEM), World Reference Base for Soil Resources (WRB), and soil texture). On the basis of Savitzky–Golay (S-G) smoothing and first derivative (FD) preprocessing of the original spectrum, three clusters were obtained by K-means clustering through the use of Vis-NIR and used as spectral classification variables. Using Vis-NIR as Model 1, Vis-NIR combined with spectral classification as Model 2, environmental variables as Model 3, and the combination of all the above variables (Vis-NIR, spectral classification, environmental variables, and soil salinity) as Model 4, a SOM content estimation model was constructed using partial least squares regression (PLSR). Using the 249 soil samples, the modeling set contained 166 samples and the validation set contained 83 samples. The results showed that Model 2 (validation r^2^ = 0.78) was better than Model 1 (validation r^2^ = 0.76). The prediction accuracy for Model 4 (validation r^2^ = 0.85) was better than Model 2 (validation r^2^ = 0.78). Among these, Model 3 was the worst (validation r^2^ = 0.39). Therefore, the combination of environmental variables with Vis-NIR spectroscopy to estimate SOM content is an important method and has important implications for improving the accuracy of SOM predictions in arid regions.

## 1. Introduction

Soil is composed of various components such as organic and inorganic minerals, microorganisms, and water. Soil organic matter (SOM) is about 1.72 times more than soil organic carbon, consists of plant and animal residues at different stages of decomposition, and is related to soil fertility and stability [1]. Rapid and accurate monitoring of SOM content is of great significance for sustainable agricultural development in arid zone saline lands [2]. Due to rapid population growth and improper land management in the last decade, water demand in arid areas has increased dramatically; this has exacerbated the conflict between agricultural and ecological water demand, and increases in areas with secondary salinization have also exacerbated land degradation [3]. This indicates that the SOM content and its spatial distribution are changing dramatically in the region, and these problems have caused governmental and public concerns about sustainable development of oasis agriculture and the ecological environment. However, SOM determination is still based on the traditional indoor analysis method (potassium dichromate—external heating method), which has problems such as large consumption of chemical reagents, large amounts of toxic waste, and time-consuming analysis in the determination of SOM, which cannot meet the needs of smart agriculture and can cause environmental pollution [4,5].Meanwhile, proximal sensing technology has the advantages of rich image information, easy data acquisition, and low cost. These factors facilitate gathering complete soil reflectance information in a short period of time. The analysis of soil properties using diffuse reflectance spectroscopy in the visible and near-infrared (Vis-NIR) wavelengths is an important topic of research today [6]. Krishnan et al. studied the spectral reflectance of selected soils in the Vis-NIR and determined the optimal wavelength for predicting the SOM content [7]; Van et al. selected a wavelength range of 1100–2500 nm for estimating the SOM content in agricultural and natural grasslands and achieved a high accuracy [8]. The potential of Vis-NIR spectroscopy in analyses of soil properties has been demonstrated in a large number of studies [9,10]. Therefore, the use of hyperspectral estimations of SOM is of great practical importance for quantifying soil fertility in arid zones.

Previously, scholars have used environmental variables to predict SOM; Hu et al. selected a total of 10 indicators such as the elevation, aspect, slope, plan curvature, and cross-sectional curvature, and topographic health index for spatial prediction of soil organic matter content by machine learning models [11], monitoring of soil properties by spectroscopic techniques in combination with auxiliary variables has been studied in some previous experiments. The strategy of combining Vis-NIR spectra with soil auxiliary information led to improvements in the performance of soil cadmium models and the accuracy of cadmium estimates [12]. When other auxiliary variables are added to the spectral data, reducing the number of spectral predictor variables is important for improving predictions [13]. Hyperspectral techniques were used to estimate SOM, and the addition of soil EC, pH, and Fe data as auxiliary variables was used to explore the potential of adding soil auxiliary information to Vis-NIR spectral models for improving the accuracy of SOM estimation models [14]. However, most of the auxiliary variables added are measured soil properties (e.g., soil EC, pH) or ions, and the measurements are expensive, time-consuming, and destructive. In the above study, we found that hyperspectral data provide high accuracy, which is relatively rare when combined with soil property datasets to estimate SOM, and its use in combination with Vis-NIR has wide application prospects.

Research on organic matter is crucial in fragile ecological environments because agricultural production is the main economic pursuit in arid areas. Specifically, the purpose of this study was to develop a predictive model of SOM using Vis-NIR spectra and auxiliary variables as covariates, and to investigate the effect of auxiliary variables on the model using partial least squares regression (PLSR) with the aim of estimating SOM more accurately and quickly (Table 1). To evaluate the relative importance of each waveband in the projection used in each of the PLSR models, variable importance in projection (VIP) scores were used to determine important wavelengths used in the PLSR calibration [15,16]. The main idea of hyperspectral inversion of SOM based on the PLSR method is to reduce spectral dimensionality while using spectral data to reveal the main controlling factors for a maximum of organic matter sampling points so that the established model is highly robust.

## 2. Materials and Methods

### 2.1. Study Area

**Figure 1:**
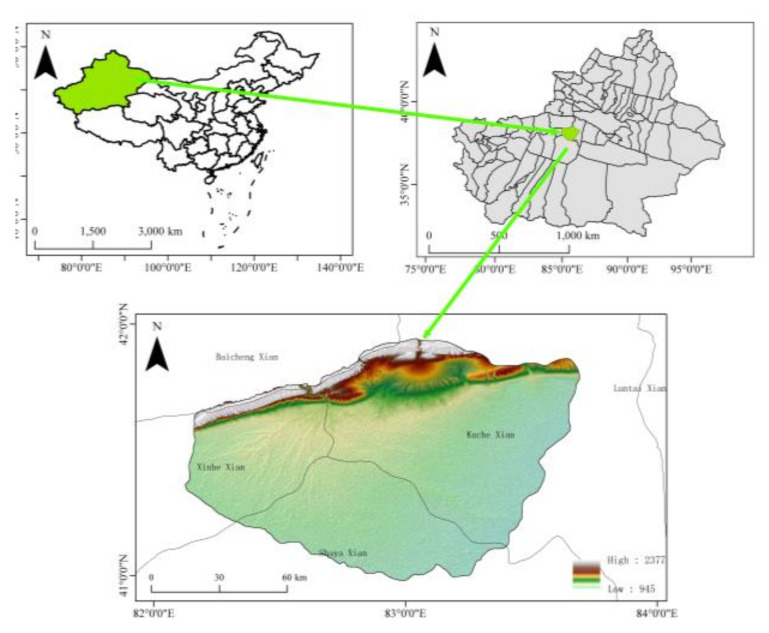
Distribution of sampling points.

The Wei-Ku Oasis is located in the northern Tarim Basin of Xinjiang, China, where the topography is high in the north and low in the south and tilts gradually from northwest to southeast; the altitude range is 830~1780 m, and the boundary is 41°05′~1°40′ N, 82°05′~83°40′ E, with a total area 52,300 km^2^ (Figure 1). The Werigan–Kuqa oasis has a typical warm temperate continental arid climate with scarce precipitation, average annual precipitation of 51 mm in the last ten years, and strong regional evaporation averaging 2700 mm annually [17]. The soil texture is mainly clay loam, chalky clay loam, loamy clay, and chalky clay. LULC mainly includes agricultural land, grassland, bareland, woodland and saline land, and natural vegetation includes *Tamarix chinensis Lour, Halostachys caspica, Kalidium foliatum, Alhagi sparsifolia Shap, Karelinia caspia, Phragmites communis*, and *Populus euphratica.*

### 2.2. Soil Sample Collection and Analysis

Collection of soil sample data was carried out in recent years. Surface layer (0–10 cm) soil was collected by the five-point cross-sampling method and mixed well into sealed bags. A total of 249 soil samples were collected, and coordinate information for the sampling points was recorded with a handheld GPS. Soil samples were dried naturally (25 °C, i.e., room temperature), ground after removal of gravel particles and vegetation by sieving, and the sieved soil divided sample equally into three parts, the first for soil spectroscopy, the second for SOM determination, and the third for soil salinity. The reflection spectrum of the soil was measured with an ASD FieldSpec 3 hyperspectrometer (Analytical Spectral Devices, Boulder, CO, USA) with a resampling interval of 1 nm, a wavelength range of 350~2500 nm, and an output band of 2151. The spectra were measured in a dark environment using a 50 W halogen lamp, a fiber optic probe with a 5° field of view, the probe is located 30 cm vertically above the surface of the soil sample, and with a 45° angle between the irradiation direction of the light source and the vertical direction [14]. The SOM content was determined by oxidation with potassium dichromate-sulfuric acid solution, and the determination standard was based on the method for determining soil organic matter (GB 9834-88). Soil salinity was determined by using a Cond7310 conductivity meter manufactured by WTW, Germany, with a water-soil ratio of 5:1, place in a conical flask and standing about 2 h, and the conductivity of the leaching solution was determined [18].

### 2.3. Spectral Data Processing

Hyperspectral data contain chemical information on the sample itself and also irrelevant information and noise, i.e., linear or nonlinear transformations of signal intensity caused by absorption and scattering on the soil surface and signal noise problems. Therefore, to solve these problems, we first removed the two spectral regions 350~399 and 2401~2500 nm from the original band and performed polynomial smoothing, S g smoothing (Savitzky–Golay smoothing), and first derivative (FD) processing of the reflectance of the original band. S-G smoothing decreased the noise to improve the signal-to-noise ratio, derivative transformation effectively eliminated interference from the baseline, other backgrounds and overlapping peaks, and improved resolution and sensitivity [19,20,21]. The volume of soil spectral data was heavy, and there was the problem of multiple correlations among the bands, so all 249 sampling points were used for dimensionality reduction by principal component analysis (PCA) after preprocessing. The contribution of the first variable obtained was PCA1 = 82%, the contribution of the second variable was PCA2 = 16%, and the cumulative contribution of the first two variables was 98%. The K-means clustering algorithm (K-means) was used to classify soil spectra with similar spectral reflectance and curve characteristics into one category. This method calculated the gap value for each cluster and selected the largest gap to classify the spectral reflectance into three categories [22].

### 2.4. Processing of Environment Variables

Soil texture and WRB data from SoilGrids250m were based on classifications of sampling points in Arcgis 10.2, and sample data were classified into the following four soil texture categories with international soil texture classification standards: clay loam (15% < clay < 25%, 20% < chalk < 40%, 30% < sand < 55%), chalky clay loam (15% < clay < 25%, 45% < chalk < 85%, 0% < sand < 40%), loamy clay (25% < clay < 45%, 0% < chalk < 45%, 10% < sand < 55%), and chalky clay (25% < clay < 45%, 45% < chalk < 75%, 0% < sand < 30%). Soil texture in ArcGIS were classified into 5 categories, which were compared to international soil classification standards and translated into domestic soil classification standards. LULC data were classified into 4 categories, agricultural land, forestland, grassland, and bareland, by combining sampling points and obtaining land use information [23]. Elevation data were obtained from the Shuttle Radar Topography Mission (SRTM) data at a resolution of 90 m (srtm.csi.cgiar.org), and the extracted elevations ranged from 949 to 1028 m for the sampling points. Salinity values ranging from 0.10 to 67.09 g/kg, ±13.52 g/kg.

### 2.5. Modeling and Accuracy Verification

PLSR is a multivariate statistical method that solves problems related to covariance, simultaneous analyses of multiple dependent variables, and studies of influence relationships when dealing with small samples. The main idea of hyperspectral inversion of soil organic matter based on the PLSR method is to reduce spectral dimensionality while using spectral data to reveal the main controlling factors for a maximum of organic matter sampling points so that the established model is highly robust [15,24].

**Figure 2:**
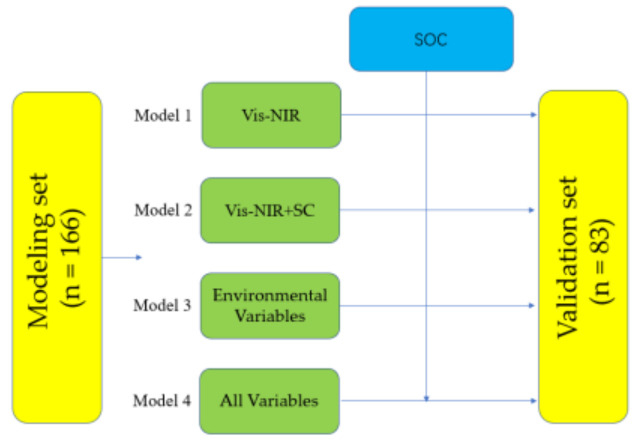
Flowchart of the study procedure.

A stratification strategy is used to divide the entire SOM dataset into modeling and validation sets, and it is an effective sample division method for modeling representative samples [25]. Specifically, 249 samples are classified according to the SOM content from small to large, and then 249 samples are divided into 83 layers, each layer containing 3 samples, the second sample assigned into validation set, and the other two samples assigned into modeling set, so the modeling dataset contains 166 samples and the validation dataset contains 83 samples (Table 2). The specific steps are shown in Figure 2. The performance of each model was evaluated by the coefficient of determination (R^2^) (Equation (1)), root mean square error (RMSE) (Equation (2)), and the ratio of performance to interquartile distance (RPIQ) (Equation (3)). RPIQ gives a good indication of nonnormal variables, RPIQ is the ratio of quartile spacing to RMSE, which is the difference between the third quartile and the first quartile of the sample; RPIQ ≥ 2.2 means the model is excellent, 1.7 ≤ RPIQ < 2.2 means the model has a balanced predictive power, and RPIQ < 1.4 means the model has a low confidence level [26].

(1) $$R^{2}=\frac{\sum {\left(y_{i}-\hat{y}\right)}^{2}}{\sum {\left(y_{i}-\bar{y}\right)}^{2}}$$

(2) $$\mathrm{RMSE}=\sqrt{\frac{1}{n}\overset{n}{\underset{i=1}{\sum }}{\left(y_{i}-\hat{y}\right)}^{2}}$$

(3) $$\mathrm{RPIQ}=\frac{\left(Q3-Q1\right)}{\mathrm{RMSE}}$$

where ${\hat{y}}_{i}$ = predicted value, $\bar{y}$ = mean observed value, $y_{i}$ = ith observed value, *n* = number of samples with *i* = 1, 2,…, *n*, Q1 = 25% of samples, Q3 = 75% of samples, and Q3 − Q1 = interquartile distance, which represents the range containing 50% of the population around the median.

The significant wavelengths in the PLSR calibration were determined by the regression coefficients (b coefficients). A wavelength was considered significant if b exceeded a threshold. Coefficient values above the threshold were considered significant, and the threshold was set as the standard deviation of its value [27,28]. To assess the relative importance of each wavelength used in each PLSR model in the projection, a VIP score was used to determine the important wavelengths used in the PLSR calibration. If a wavelength had a VIP score above 1, that wavelength was considered an important wavelength [29,30]. In this study, we calculated VIP scores for the respective variables to determine their importance.

S-G smoothing and FD processing were performed in Unscrambler X 10.4 software (Oslo Science Park Gaustadalléen, Oslo, Norway), classification of environmental covariates was performed in ArcGIS 10.2 software (Contractor/Manufacturer is Esri, 380 New York Street, Redlands, CA, USA), statistical analysis and plotting of data were performed in MATLAB R2012a (MathWorks, Natick, MA, USA) and Origin 2021 software (Version 2021. OriginLab Corporation, Northampton, MA, USA), respectively, and PLSR modeling validation was performed in Unscrambler X 10.4 software.

## 3. Results

### 3.1. Descriptive Statistics of Soil Properties

The descriptive statistics of soil properties (Table 3) show that the maximum value of SOM in the samples was 31.70 g kg^−1^, the minimum value was 0.31 g kg^−1^, and the coefficient of variation was 61.47%, indicating that the organic matter content varied widely at sampling sites and SOM was relatively discrete; the maximum value for salt was 67.09 g kg^−1^, and the minimum value was 0.10 g kg^−1^, indicating that the salt content of the sampling sites varied widely; the coefficient of variation was 97.63%, indicating a medium variation.

### 3.2. Characteristics of Reflection Spectral Curves under Each Auxiliary Variable Classification

**Figure 3:**
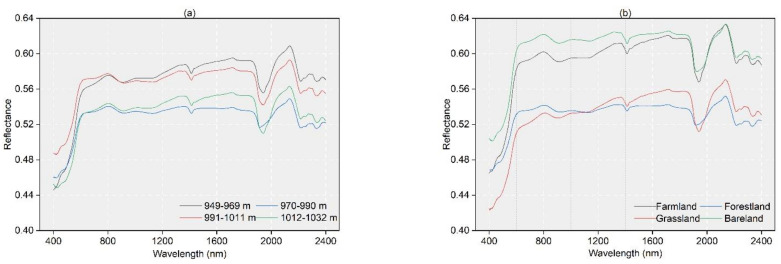

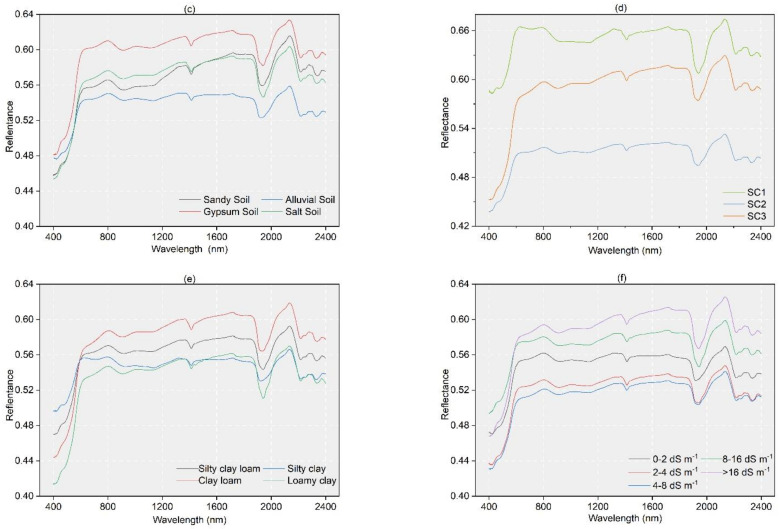
Spectral reflectance data with different auxiliary variables; (**a**) DEM, (**b**) LULC, (**c**) soil texture, (**d**) spectral classification, (**e**) WRB, and (**f**) salinity.

Soil reflectance and spectral characteristics depend on the classification level of environmental variables (Figure 3), and the three main absorption features were located at approximately 1400, 1900, and 2200 nm. The absorption feature at 1400 nm, which is a representative absorption region for water, combined O-H bending and stretching vibrations of free water, and the combined features at approximately 1900 and 2200 nm were due to bending and stretching vibrations of Al-OH and Mg-OH and the presence of clay mineral absorption bands, respectively [30,31].

**Figure 4:**
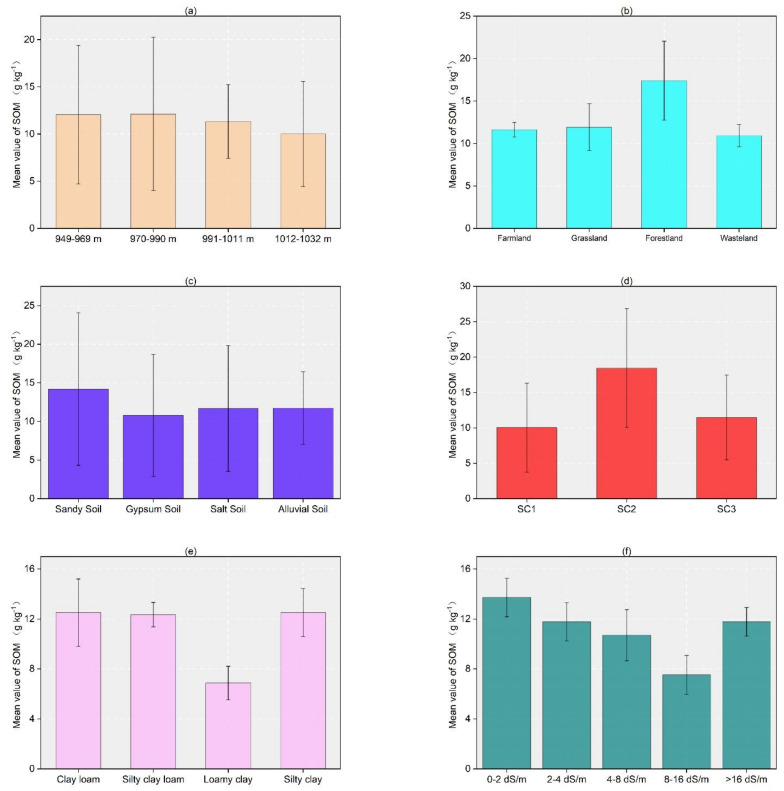
Mean SOM contents for each auxiliary variable and their standard errors; (**a**) DEM, (**b**) LULC, (**c**) soil texture, (**d**) spectral classification, (**e**) WRB, and (**f**) salinity (black lines represent standard errors).

Similar shapes were observed for soil spectral curves with different auxiliary variables. It is worth noting that there were significant differences among spectral reflectance data for each auxiliary variable. The reflectance data for the soil showed decreasing trends with increasing elevation in the DEM classification (Figure 3a). The higher SOM content in farmland and grassland in the LULC classification makes them less reflective than forestland and bareland, although the large difference in forestland and bareland reflectance compared to that of farmland and grassland may, on the one hand, be due to the small sample size, which does not cover all variations in the area; on the other hand, we cannot ignore that it may be due to the lack of precise LULC classification (Figure 3b). From Figure 3d and Figure 4d, it can be seen that the overall trend of soil spectral reflectance decreases with the increase in soil organic matter content. Among soil textures, clay loam showed the highest reflectance in the range 1000 to 2400 nm, and the difference in reflectance between silty clay and loamy clay was smaller (Figure 3e). In the salinity classification, there were significant differences among the reflectance values for each salinity (Figure 3f). There were clear differences between gypsum and alluvial soils and two other types of soils in the WRB (Figure 3c).

### 3.3. SOM and Standard Error with Each Auxiliary Variable Classification

There are some differences in the SOM values in the auxiliary variable minutes (Figure 4). Except for SOM values for the elevation interval 1012 to 1032 m (DEM) 10.01 g/kg, those for gypsum soils (WRB group) 10.79 g/kg, loamy clay soils (soil texture) 6.88 g/kg, moderate salinity (salinity) 10.71 g/kg, and the first spectral class (spectral classification) 10.05 g/kg were significantly lower than the overall SOM values of samples. Higher SOM values are associated with forestland 17.41 g/kg, sandy soil 14.19 g/kg, clay loam 12.52 g/kg, silty clay loam 12.35 g/kg, silty clay12.52 g/kg, nonsaline 13.73 g/kg, strongly saline 11.78 g/kg, and second spectral class soil 18.46 g/kg. The DEM data were divided into four gradients, SOM showed an overall decreasing trend with increasing altitude. Among the four groups, the third group had the smallest standard error 3.9 (Figure 4a). Regarding LULC, the SOM contents decreased in the order forestland 17.41 g/kg, grassland 11.92 g/kg, farmland 11.63 g/kg, and bareland 10.94 g/kg, and the standard error was smallest for farmland and largest for forestland (Figure 4b). In the WRB, the soils at the sampling sites were divided into four types, sandy, gypsum, salt, and alluvial soils, with the highest SOM content for sandy soils 14.19 g/kg and the lowest for gypsum soils 10.79 g/kg; the standard errors for the four soils were all large (Figure 4c). In the classification of soil texture, the difference between SOM contents of clay loam 12.52 g/kg, silty clay loam 12.35 g/kg, and silty clay 12.52 g/kg were small, while the difference between SOM contents for loamy clay 6.88 g/kg and the other three categories were large, and the standard errors for SOM contents with all four types of soils were small (Figure 4e). The sampling points were divided into five gradients according to salinity, and in the first four gradients, the SOM content decreased gradually as the salinity increased; in the grouping with the highest salinity, the SOM content increased again (Figure 4f).

### 3.4. Plot of PCA under Each Auxiliary Variable

**Figure 5:**
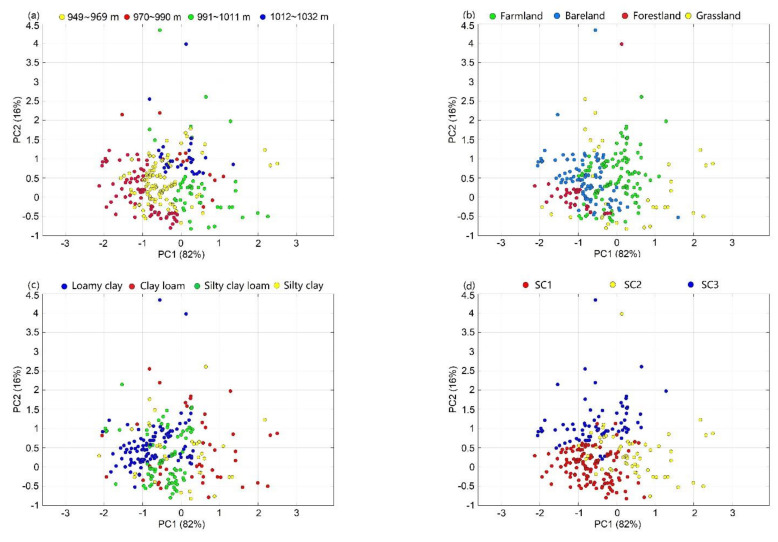

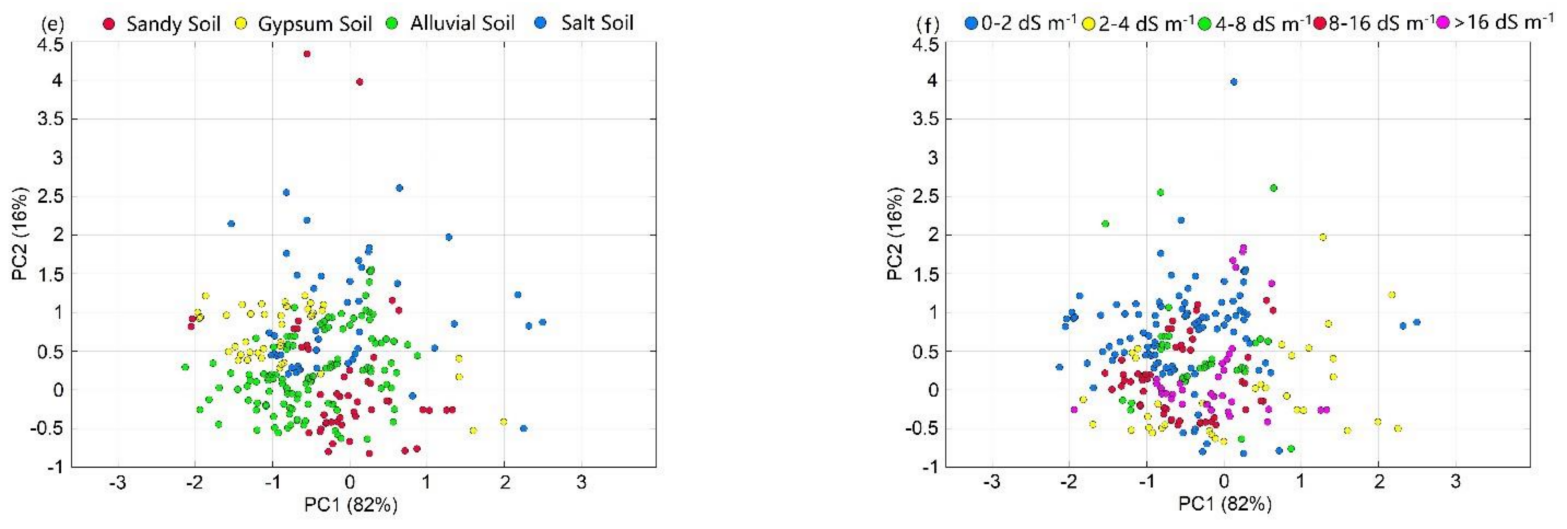
PCA results for each auxiliary variable; (**a**) DEM, (**b**) LULC, (**c**) soil texture, (**d**) spectral classification, (**e**) WRB, and (**f**) salinity.

A double-labeled plot of the first principal component (PC1) and the second principal component (PC2) for the soil spectrum was used to visualize similarities between soil samples from different spectral subbanks, and latent variable analysis of the PCA showed that PC1 + PC2 comprised 98% of the spectral variance (Figure 5). From Figure 5d, it can be found that the spectral classification is mainly based on the values of PCA1 in order of magnitude, that is, according to the magnitude of the intensity of the reflected spectrum. Then, it is based on the shape characteristics of the spectral lines, which is reflected in the differences of PCA2 values in the three clustering centers, with the PCA2 values of SC1 and SC2 basically around 0, while the value of SC3 is mainly around 1. There were some similarities among distributions of the classifications and the samples, with, for example, silty clay loam in the first category of the spectral classification and soil texture, 949~969 m in the DEM classification, and forestland in the LULC classification. On the other hand, SC3 and loamy clay, farmland, and nonsaline soils were related.

Model 3 added only environmental covariates for prediction and had the worst prediction effect, followed by Model 1, which used only spectral reflectance as a covariate for prediction. Model 4 showed better prediction accuracy compared to other models after adding environmental variables such as LULC, DEM, WRB, soil texture, and salinity, with R^2^ improved to 0.86 and RMSE = 3.85, RPIQ = 2.34. Model 2 showed a small improvement in prediction accuracy compared to Model 1 after adding spectral classification as a covariate, and it was better than Model 3.

### 3.5. Soil SOM Modeling Based on Soil Spectra and Each Auxiliary Variable

**Figure 6:**
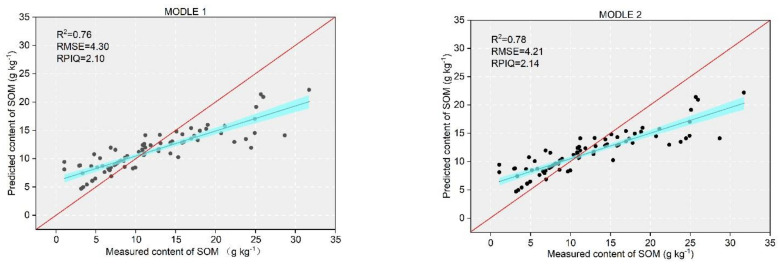

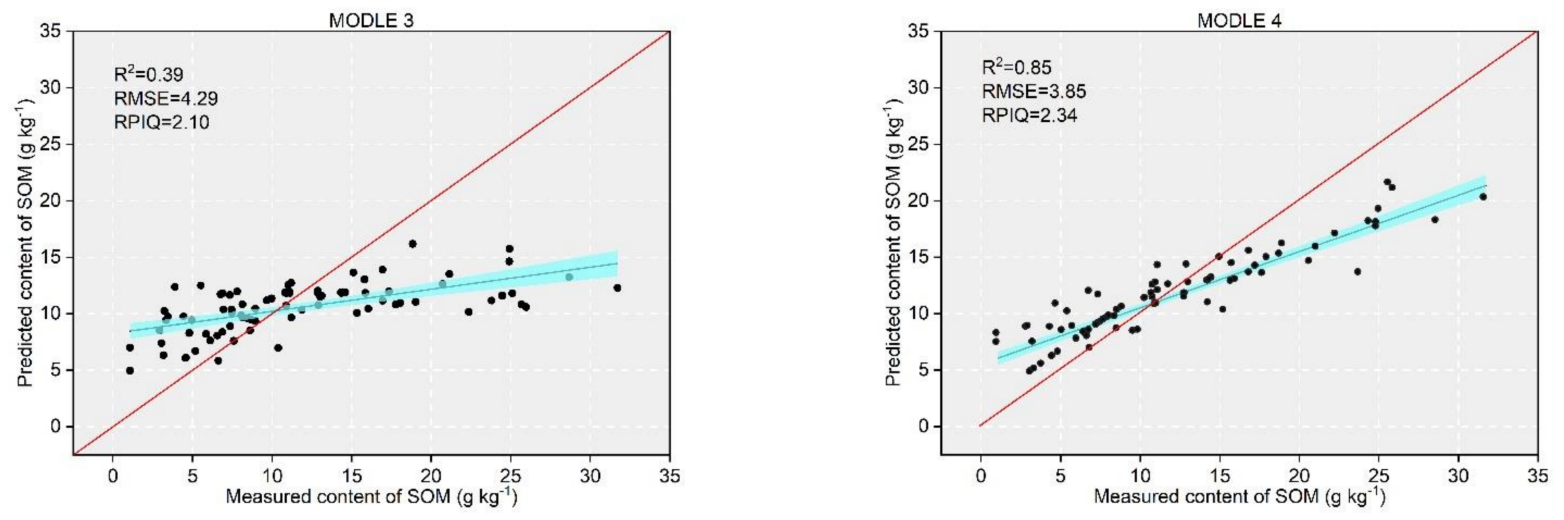
SOM models using the validation dataset (the blue area in the figure is the 95% confidence interval, the red line is the 1:1 line, and the black line is the fitted line) Model 1, SOM ~ hyperspectral; Model 2, SOM hyperspectral + spectral classification; Model 3, SOM ~ environmental variables; and Model 4, SOM ~ hyperspectral + spectral classification + environmental variables.

As shown in Figure 6, the fitted line for Model 3 had the largest angle between the fitted line and the 1:1 line, which indicated the largest and worst fit; Model 1 and Model 2 exhibited similar fits that were better than that of Model 3, and Model 4 exhibited the smallest angle between the fitted line and the 1:1 line and showed the best fit.

### 3.6. Importance Analysis of Auxiliary Variables in the Model

**Figure 7:**
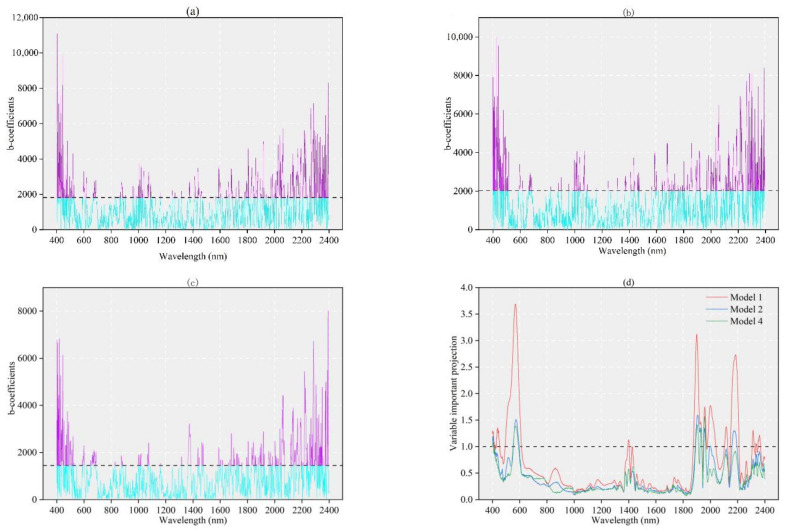

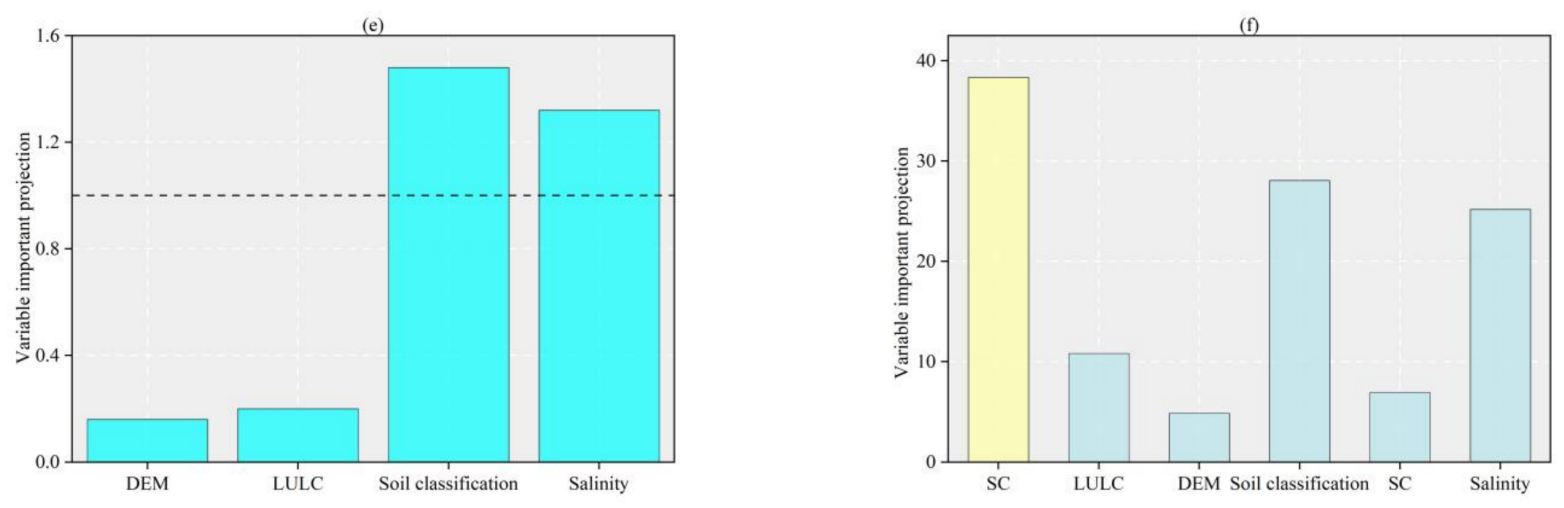
VIP scores for the b-coefficients and environmental covariates based on PLSR cross-validation models; (**a**–**c**) show the b-coefficients, in which the dashed lines are the thresholds for the b-coefficients, and (**a**) = 1813.84, (**b**) = 2019.01, and (**c**) = 1447.63. (**d**–**f**) show VIP scores for the auxiliary variables, and the dashed line is the critical value of the VIP score; (**a**) Model 1, (**b**) Model 2, (**c**) Model 4, (**d**) scores of spectra in Model 1, Model 2, and Model 4, (**e**) scores of auxiliary variables in Model 3, and (**f**) scores of auxiliary variables in Model 2 and Model 4. (First yellow bar represents SC in model 2 and the fifth blue bar represents SC in model 4.) SC-Spectral classification.

The distributions of b-coefficients (Figure 7a–c) over the entire spectral wavelength range 400–2400 nm showed several different wavelength bands for soil properties, and the magnitudes of the coefficients represented the importance of wavelength in explaining variations in soil properties. b-coefficient distributions were similar for the three models and, although the magnitude of b-coefficients changed in some bands, most of the important bands remained the same; for example, b-coefficients exhibited clear peaks in both the visible and near-infrared bands, and the main important wavelengths were 400–520 nm, 990–1040 nm, 1960–2080 nm, 2120–2250 nm, and 2300–2400 nm.

The VIP scores of spectral and auxiliary variables are shown in Figure 7d–f, and the values of VIP scores > 1.0 in the visible and near-infrared bands in the spectral scores of Model 1, Model 2, and Model 4 occurred at approximately 490–600 nm, 1890–2100 nm, and 2200–2280 nm; the main peaks occurred at approximately 530 nm, 1900 nm, 1980 nm, and 2250 nm in Model 2 and Model 4; the environmental covariate scores are shown in the figure; the covariate scores were greater than 1 in both Model 2 and Model 4; and spectral classification had high contributions in Model 4. After adding environmental covariates, the contribution of spectral classification decreased, the highest contributions were made by soil classification and salinity, and the contribution of LULC was higher than that of DEM; however, in Model 3, the scores of environmental variables were both lower, probably because Model 3 did not add spectra as variables for modeling predictions, so the contributions were smaller. Only the scores for soil classification and salinity were greater than 1 in Model 3, and the scores for LULC and DEM were less than 1, indicating that soil classification and salinity had higher contributions to the model, and the contributions of LULC and DEM were lower. This may have had two causes; on the one hand, the elevation differences in the study area were small, and the differences among sampling points after classification were also small. On the other hand, the land use classifications in the study area were not fine, resulting in imprecise classifications of some points.

## 4. Discussion

Changes in SOM in the research area were associated with changes in environmental (LULC, DEM) and soil (soil texture, WRB, and salinity) covariates. In all covariate groups, the majority of samples had SOM contents of 12–14 g/kg, and LULC, DEM, WRB, soil texture, and salinity had strong effects on changes in SOM (Figure 4), thus indicating that these were the main factors affecting the overall SOM in the study area.

All of the high SOM predictions were biased, with the largest difference in predictions for Model 1 and the closest predictions for Model 4 (Figure 6), indicating that the use of auxiliary variables in Model 4 had a significant effect on improving the SOM predictions. Our results indicated that combining auxiliary variables and Vis-NIR reflectance improved the SOM content prediction accuracy, with a small increase in the accuracy of the spectral-like covariates observed in Model 2 and a decrease in the RMSE of only 0.09 compared to Model 1 (Figure 6); this suggested that, in the absence of other environmental and soil covariates, the use of this covariate may improve the accuracy of the prediction model. In this study, despite the good model performance in estimating SOM, 15% of the SOM variation information was not captured (Table 4). For the SOM content prediction model, the inclusion of these auxiliary variables improved the prediction accuracy (Figure 7). In both Model 1 and Model 2, the inclusion of spectral classification improved the prediction accuracy and, although spectral classification had the highest VIP score in Figure 7f, Model 2 only improved the r^2^ value by 0.2 and reduced the RMSE by 0.09 compared to Model 1. This indicates that using spectral classification as a separate variable without combining it with other auxiliary variables also improved the accuracy of the model, which is consistent with the results of previous studies [32,33].

Compared with Model 1, Model 3 and Model 4 showed a reduction in the RMSE of 0.01 and 0.45, respectively, after the inclusion of relevant environmental variables, and the largest contribution of soil classification and salinity in this study (Figure 7), which is consistent with Wang’s study [34]. The higher importance of the salinity content in the model predictions was reflected in two aspects; on the one hand, salinity and water in the subsoil easily penetrate into the topsoil due to capillary action; on the other hand, increased soil moisture is more conducive to the growth of saline plants and the production and decomposition of apoplastic matter [35]. Therefore, the SOM content increases [27,36]. Although both LULC and DEM factors are less important for model prediction, they are still the main factors affecting SOM [37].

The low importance of LULC may be due to two reasons. First, agricultural management, such as tillage, leads to the degradation of some organic matter in the soil, which accelerates the decomposition and loss of SOM, which also increases the prediction bias, and the second point may be the coarse resolution of the environmental variables themselves, which makes their classification imprecise and leads to their low importance, and we will include higher resolution data in future studies to reduce this error [7].

The b-coefficient of SOM has a clear peak in both of the Vis-NIR. The PLSR model of SOM has significant wavelengths at approximately 450–520, 600–650, 900–1150, 1470, 2100, 2250, and 2300 nm (Figure 7a). The first four wavelengths in the visible region were identified as particularly important for the SOM. This is in agreement with previous findings that the visible wavelengths of 486, 607, and 650 nm are important for SOM prediction [38,39]. Dalal et al. found 1700 nm and 2050 nm to be important for SOM prediction [40] and 1726–2426 nm for SOM identification [41]. The spectral band at 1340~1380 nm is mainly associated with carbon-hydrogen bonds (C-H), while the bands at 1860~1900 nm are mainly associated with nitrogen-hydrogen (N-H) and oxygen-hydrogen (O-H) bonds. Moreover, the 2200–2220 nm band includes phenolic O-H, amide N-H, amine N-H, and aliphatic C-H [42,43].

In previous studies, environmental variables have been combined with machine learning methods to estimate soil organic matter [11], and soil organic matter models have also been developed by combining spectral and spectral classification with partial least squares [31]. However, in this study, using both auxiliary variables and spectral information as input variables was significantly better than using auxiliary variables or spectral information alone as input variables, indicating that combining the two can improve the prediction accuracy of soil organic matter.

The Wei-Ku Oasis is a typical arid and semiarid region. This study enriches the study of SOM regarding Vis-NIR spectra and provides a new perspective for estimating the SOM of desert soils by adding auxiliary variables.

Due to the unique regional and territorial natures of soils in different regions, it is possible that our study area is in an arid and semiarid region with more severe salinization, and the high or low salt contents affect the reflectance of the soil and influence the accuracy of the model. Therefore, in the future, there is a need to develop a chemometric approach to reduce salinity effects by pre-processing the data, thus improving the model quality and achieving the expected fast estimation [44]. Soil properties, which are rich in soil information, are obtained with multiple analytical tools or multiple measurement platforms used to detect and measure the same samples simultaneously [35]; this combines middle infrared (MIR) and Vis-NIR data to improve the estimation of SOM, or the implementation of multisensor data fusion is also a method in which different aspects of soil variability can be combined [36]. Likewise, we should focus on more local- and regional-scale soil observations. As most of the uncertainty in SOC prediction is attributed to local- and regional-scale variability [45], our study is based on past data to predict SOM; it is also worth pondering how to use data from real-time dynamic monitoring to predict soil SOM contents in the present or future.

## 5. Conclusions

In this research, the effects of different combinations of Vis-NIR spectra, spectral classification, and environmental factors on the accuracy of SOM predictions were evaluated. The results showed that:

1. Spectral classification exhibited significant differences in grouping reflectance curves and SOM contents, while the differences among other variables were small; therefore, spectral classification better distinguished the reflectance spectral data and maximized the variability of spectral characteristics for different SOM contents.
2. The addition of the environmental factor to the spectral variables provided an effective predictive variable for estimation of SOM, which greatly improved the prediction accuracy of the model; the R^2^ was improved from 0.78 to 0.85.
3. Spectral information played an important role in predictions of SOM contents.

In addition, among the selected environmental factors, soil classification and soil salinity also played important roles in predicting SOM content; this provides a basis for improving the accuracy of SOM estimation and screening of more effective predictors for arid and semiarid regions, and it is of great significance for guiding agricultural production in arid regions.

## Figures and Tables

**Table 1:** All variables in this study.

Environmental Variables	Vis-NIR Spectroscopy	Spectral Classification
Land-use/land-cover (LULC)	Vis-NIR	SC1
Digital elevation model (DEM)		SC2
World Reference Base for Soil Resources (WRB)		SC3
Soil texture		
Soil salinity		

**Table 2:** Variable sets for each model.

Models	Input Variables
Model 1	Vis-NIR
Model 2	Vis-NIR, Spectral classification
Model 3	DEM, LULC, WRB, Soil salinity, Soil texture
Model 4	Vis-NIR, Spectral classification, DEM, LULC, WRB, Soil salinity, Soil texture

**Table 3:** Statistical descriptions of SOM and other soil properties.

Attributes	Sample Sets	Na	Min	Max	Median	Mean	SD ^b^	CV ^c^ (%)
SOM (g kg^−1^)	Entire ^a^	249	0.31	31.70	10.88	11.87	7.28	61.47%
	Calibration	168	0.31	31.06	11.08	11.90	7.42	62.32%
	Validation	83	1.07	31.70	10.82	11.80	6.99	59.24%
Salinity (dS m^−1^)	Entire	249	0.10	67.09	12.29	13.90	13.57	97.63%

^a^ Sample numbers; ^b^ Standard deviation; and ^c^ Coefficient of variation.

**Table 4:** Prediction accuracy of the SOM validation set using different models.

Models	Lv ^a^	R^2^	RMSE ^b^	RPIQ ^c^
Model 1	7	0.76	4.30	2.10
Model 2	8	0.78	4.21	2.14
Model 3	1	0.39	4.29	2.10
Model 4	13	0.85	3.85	2.34

^a^ Latent variable; ^b^ Root mean square error; ^c^ Ratio of performance to interquartile distance.

## Data Availability

Not applicable.
